# Hypothyroidism is associated with worse outcomes of hepatocellular carcinoma patients after liver transplantation

**DOI:** 10.1002/cam4.1797

**Published:** 2018-11-19

**Authors:** Ning Zhang, Weidong Jin, Shuangnan Zhou, Ju Dong Yang, William S. Harmsen, Nasra H. Giama, Nicha Wongjarupong, Julie K. Heimbach, Kymberly D. Watt, Harmeet Malhi, Terry M. Therneau, Lewis R. Roberts

**Affiliations:** ^1^ Division of Gastroenterology and Hepatology Mayo Clinic College of Medicine and Science Rochester Minnesota; ^2^ Integrated TCM & Western Medicine Department 302 Military Hospital Beijing China; ^3^ Department of General Surgery Wuhan General Hospital of PLA Wuhan China; ^4^ Liver Transplantation Center 302 Military Hospital Beijing China; ^5^ Division of Biomedical Statistics and Informatics Mayo Clinic College of Medicine and Science Rochester Minnesota; ^6^ Department of Surgery Mayo Clinic College of Medicine and Science Rochester Minnesota

**Keywords:** hypothyroidism, liver cancer, orthotopic liver transplantation, outcomes, risk factors

## Abstract

**Background/Aims:**

Hypothyroidism has been associated with hepatocellular carcinoma (HCC) incidence; however, the relationship between hypothyroidism and HCC patient outcomes is unclear. We investigated the impact of hypothyroidism on outcomes after liver transplantation for HCC.

**Materials and Methods:**

We retrospectively studied HCC patients transplanted between January 2000 and December 2015. Hypothyroidism was defined as a thyroid‐stimulating hormone (TSH) level continuously greater than 5 mIU/L, a documented history of hypothyroidism, or treatment with thyroid hormone replacement therapy. Multivariate Cox regression was used to assess the impact of hypothyroidism on overall survival (OS) and recurrence‐free survival (RFS) adjusting for potential confounders. Subgroup analyses and interaction tests were conducted to compare the impact of hypothyroidism in different subgroups and assess for possible synergistic effects. Sensitivity analyses were performed among different cohorts to verify the stability of the results.

**Results:**

A total of 343 HCC patients who underwent liver transplantation were included in the analysis. The primary analysis was conducted among 288 patients diagnosed with HCC prior to transplantation. Hypothyroidism was independently associated with worse OS and RFS, as was elevated TSH. The adjusted hazard ratio (AHR) of hypothyroidism was 2.45 (95% confidence interval [CI], 1.44‐4.18) for OS and 5.54 (2.36, 13.01) for RFS. The AHR of TSH for OS was 1.05 (1.02, 1.09) and 1.08 (1.03, 1.13) for RFS. No interaction was found among different subgroups categorized by etiology and comorbidity. The results were stable to sensitivity analyses.

**Conclusion:**

Hypothyroidism is associated with poorer overall and recurrence‐free survival of HCC patients receiving liver transplantation. These results require validation.

## 1 INTRODUCTION

Hepatocellular carcinoma (HCC) is the major form of primary liver cancer, which is the sixth most common cancer and the second leading cause of cancer‐related death globally.^1^ Liver transplantation is the most effective first‐line curative option for HCC patients diagnosed at early stages.^2^ Due to the shortage of donor organs, it is important to identify novel factors associated with the outcomes of patients receiving liver transplantation for HCC, as this may help optimize screening and selection of patients for liver transplantation and establish effective strategies to improve the overall prognosis. This is particularly important because of the changing secular trends in the causative etiologies of HCC, from the dominant etiologies of chronic viral hepatitis B and C to the increasing proportion of metabolic syndrome‐related liver diseases such as nonalcoholic fatty liver disease (NAFLD) and nonalcoholic steatohepatitis (NASH).^3^

Hypothyroidism has been reported to be related to several kinds of cancers. It was reported to be associated with a higher incidence of breast cancer and prostate cancer^4^, ^5^ and also as a side effect among patients with neck cancer, breast cancer and renal cancer receiving radiotherapy or molecular‐targeted therapy.^6^, ^7^ Moreover, the development of secondary hypothyroidism was observed to be associated with better survival.^8^, ^9^, ^10^, ^11^, ^12^ In HCC patients, we have previously found hypothyroidism to be significantly more prevalent in patients with HCC of unknown etiology.^13^ Another study showed that hypothyroidism was associated with a 2.8‐fold higher risk of incidence of HCC in women.^14^ An animal study suggested that hypothyroid status favored the onset and progression of pre‐neoplastic lesions to HCC.^15^ However, it is still not clear how hypothyroidism affects the prognosis of HCC patients.

In this retrospective study, we aimed to investigate the influence of hypothyroidism diagnosed before the onset of HCC on patient outcomes after liver transplantation.

## 2 MATERIALS AND METHODS

### 2.1 Study population

We included patients aged over 18 years old who were diagnosed with HCC and treated by liver transplantation at the Mayo Clinic, Rochester, MN between January 2000 and December 2015 in this retrospective cohort study. We identified eligible HCC cases from the Mayo Clinic Advanced Cohort Explorer (ACE) platform using the International Classification of Diseases (ICD) codes of ‘‘155.0’’ and “c22.0” and the keywords ‘‘hepatocellular carcinoma” and “explant” or “orthotopic liver transplantation (OLT)” or “donor” or “receipt” in pathology reports to identify the cases treated by liver transplantation. We excluded patients diagnosed with cholangiocarcinoma (CCA), mixed HCC and CCA or fibrolamellar HCC based on explant pathology. The study was approved by the Mayo Clinic Institutional Review Board.

The diagnosis of HCC was based on either noninvasive criteria (a new mass >1 cm in size developing in a cirrhotic liver and characterized by arterial enhancement and portal venous washout on computed tomography [CT], magnetic resonance imaging, or angiography) or histopathology from the biopsy or the explanted liver, using the diagnostic criteria of the American Association for the Study of Liver Disease (AASLD) 2010 Guidelines for HCC.^16^ All patients met UNOS criteria for listing for liver transplantation. The primary outcomes were overall survival (OS) and recurrence‐free survival (RFS). Subjects who were alive or lost to follow up were censored at the date of last contact.

### 2.2 Data collection

Demographic characteristics, medical history, smoking status, laboratory results, underlying liver diseases, and tumor characteristics from the explanted liver were collected. Comorbidities before the diagnosis of HCC, including diabetes, hypertension, hyperlipidemia, hypercholesterolemia, hypothyroidism, and coronary artery disease were abstracted based on the corresponding ICD‐9 and ICD‐10 codes and confirmed by review of the clinical notes. Hypothyroidism was defined as a thyroid‐stimulating hormone (TSH) level continuously >5 mIU/L, a documented physician diagnosis in the medical record or the use of thyroid hormone replacement therapy.^13^ Subjects diagnosed with hypothyroidism secondary to the treatment of hyperthyroidism were not included in the study. Underlying liver diseases were ascertained by positive HBsAg for hepatitis B virus (HBV) infection, positive HCV RNA or anti‐HCV for hepatitis C virus (HCV) infection, history of excessive alcohol use for alcoholic liver disease (ALD), metabolic syndrome combined with steatosis for NAFLD, and serological and/or histopathologic diagnosis for autoimmune liver diseases (AILD) comprising autoimmune hepatitis, primary biliary cholangitis, primary sclerosis cholangitis, and others. Obesity was diagnosed when the body mass index (BMI, calculated as weight in kilograms divided by height in meters squared) was equal to or greater than 30.

Patients were followed up for death and recurrence of HCC from the time of liver transplantation to December 31, 2016. The Accurint system was used to determine the final vital status for patients who were lost to follow up.

### 2.3 Statistical analysis

Univariate analyses were performed using Cox proportional hazards regression to explore possible risk factors associated with HCC outcomes in patients treated by liver transplantation. Multivariate Cox proportional hazards regression analyses were performed to (a) identify the independent impact of hypothyroidism and TSH on OS and RFS after liver transplantation by adjustment for potential confounders; and (b) evaluate the interaction of hypothyroidism with different variables.

The main analysis was performed on the group of patients diagnosed with hypothyroidism before HCC diagnosis. Sensitivity analyses were conducted to evaluate the impact of hypothyroidism by (a) removing the subjects who died within 1 month after liver transplantation because they were likely to have died of operative complications (n = 5); (b) assuming that the 24 subjects whose TSH was greater than 5 mIU/L on a single occasion before transplantation, the two patients who were diagnosed with hypothyroidism after the diagnosis of HCC but before liver transplantation, and the four patients diagnosed with hypothyroidism after liver transplantation, all had hypothyroidism (n = 30); and (c) repeating the main analyses among the whole cohort including individuals found incidentally to have HCC at the time of liver transplantation (n = 343).

The hazard ratio (HR) or adjusted HR (AHR) and 95% confidence interval (CI) values of the variables were calculated based on the maximum likelihood estimation. *P* < 0.05 was regarded as the threshold for significance.

## 3 RESULTS

### 3.1 Participant characteristics

There were 343 patients eligible for the final study after excluding three patients diagnosed as CCA, 1 with mixed HCC and CCA, 12 without follow‐up information, and four who received liver transplantation outside of Mayo Clinic. Of the included participants, 55 were diagnosed with HCC incidentally based on explanted liver pathology Table S1. Considering the purpose of our study, we conducted the main analysis focusing on the group diagnosed with HCC before liver transplantation (n = 288). By the cutoff date of December 31, 2016, the median follow‐up was 7.7 years for the entire group of 343 cases and 7.8 years for the 288 patients with HCC diagnosed prior to liver transplantation.

### 3.2 Baseline characteristics of pre‐diagnosed HCC patients

To identify the key covariates contributing to the association of hypothyroidism with outcome in liver transplant recipients, we compared the baseline characteristics of HCC patients with hypothyroidism to the characteristics in patients without hypothyroidism Table 1. Among the 288 pre‐diagnosed HCC patients, 35 were diagnosed with hypothyroidism before the onset of HCC. In the group of HCC patients with a history of hypothyroidism, in addition to more frequent events of death and recurrence, there were more females, higher rates of hypertension and obesity, and higher TSH levels before liver transplantation.

**Table 1:** Baseline characteristics of patients diagnosed with hepatocellular carcinoma before liver transplantation

Variables	No hypothyroidism N = 253	Hypothyroidism N = 35	*P*
Gender = male (%)	193 (76.3)	17 (48.6)	0.001
Age (median [IQR])	58.3 (54.1, 63.1)	59.3 (57.1, 62.8)	0.132
Race = white (%)	204 (86.8)	30 (96.8)	0.190
BMI (mean [SD])	28.97 (5.37)	29.84 (4.62)	0.361
CAD = yes (%)	42 (16.6)	7 (20%)	0.794
HCSL = yes (%)	22 (8.7)	1 (2.9)	0.389
DM = yes (%)	100 (39.5)	14 (40.0)	1.000
HTN = yes (%)	82 (32.4)	21 (60.0)	0.003
HLD = yes (%)	45 (17.8)	10 (28.6)	0.196
Obesity = yes (%)	93 (36.8)	20 (57.1)	0.033
Smoking (%)			
Current	64 (25.3)	7 (20.0)	0.695
Former	63 (24.9)	8 (22.9)	
Never	126 (49.8)	20 (57.1)	
HCV = yes (%)	130 (51.4)	20 (57.1)	0.646
ALD = yes (%)	82 (32.4)	10 (28.6)	0.792
NAFLD = yes (%)	46 (18.2)	7 (20.0)	0.978
HBV =yes (%)	20 (7.9)	0 (0.0)	0.171
AILD = yes (%)	22 (8.7)	4 (11.4)	0.830
Other etiology = yes (%)	18 (7.1)	2 (5.7)	1.000
AFP (median [IQR])	8.00 (3.80, 23.00)	8 (5.55, 30.00)	0.326
TSH (median [IQR])	2.30 (1.40, 3.50)	4.00 (1.60, 7.05)	0.006
CTP score (mean [SD])	7.63 (2.16)	7.43 (2.36)	0.613
MELD (mean [SD])	14.52 (7.70)	14.31 (7.19)	0.879
Milan criteria = within (%)	157 (62.1)	21 (60.0)	0.961
Vascular invasion (%)			
No	228 (90.1)	29 (82.9)	0.313
Differentiation (%)			
Well and moderate	111 (45.1)	11 (32.4)	0.695
Poorly and undifferentiated	80 (32.5)	15 (44.1)	
No viable tumor	55 (22.4)	8 (23.5)	
Tumor number (%)			
Solitary	111 (45.1)	11 (32.4)	0.313
Two	80 (32.5)	15 (44.1)	
More than two	55 (22.4)	8 (23.5)	
Tumor diameter (%)			0.977
<3 cm	167 (69.3)	23 (69.7)	
3‐5 cm	57 (23.7)	8 (24.2)	
>5 cm	17 (7.1)	2 (6.1)	
>3 cm	74 (30.7)	10 (30.3)	1
Bridge treatment	227 (89.7)	32 (91.4)	0.988
Final status = deceased (%)	71 (28.1)	17 (48.6)	0.023
Recurrence (%)	28 (11.1)	9 (25.7)	0.031

AILD, autoimmune liver disease; ALD, alcoholic liver disease; BMI, body mass index; DM, diabetes mellitus; HBV, hepatitis B virus; HCSL, hypercholesterolemia; HCV, hepatitis C virus; HLD, hyperlipidemia; HTN, hypertension; MELD, model for end‐stage liver disease; NAFLD, nonalcoholic fatty liver disease; TSH, thyroid‐stimulating hormone.

We identified an additional 30 patients with an equivocal diagnosis of hypothyroidism Figure S1; of these, 24 patients had a single abnormal TSH measurement greater than 5 mIU/L prior to liver transplantation, two patients were diagnosed with hypothyroidism after HCC diagnosis but before liver transplantation, and four patients were diagnosed with hypothyroidism after liver transplantation. In the sensitivity analysis, we added this group of patients to those with definite hypothyroidism diagnosed prior to HCC diagnosis as Hypothyroidism‐2.

### 3.3 Factors associated with overall survival and recurrence‐free survival

Based on the previous literature and our study objectives, we examined the effects of age, gender, race/ethnicity, cirrhotic status, BMI, comorbidities before the onset of HCC, laboratory results including serum TSH, serum AFP, neutrophil to lymphocyte ratio (NLR), Child‐Turcotte‐Pugh (CTP) score, model for end‐stage liver disease (MELD) score before liver transplantation, and tumor characteristics from the explanted liver pathology on OS and RFS by univariate Cox regression analysis. We then adjusted those variables with *P* < 0.10 in multivariate Cox regression analysis to assess the independent impact of hypothyroidism and TSH on OS and RFS. The purpose that *P* < 0.10 was chosen as the criterion for inclusion of confounding factors instead of 0.05 was to incorporate as many confounding factors as possible to better demonstrate the independent significance of variables of interest.

Variables with *P* < 0.10 related to OS by univariate analysis included hypothyroidism, TSH, cirrhosis status, NLR, AFP, MELD, vascular invasion, tumor diameter, and Milan criteria. Variables with *P* < 0.10 related to RFS by univariate analysis included hypothyroidism, TSH, AILD, CTP, vascular invasion, tumor diameter, and Milan criteria. Of the comorbidities examined, only hypothyroidism was found to be independently associated with worse outcome Table 2.

**Table 2:** Factors associated with overall survival and recurrence‐free survival of hepatocellular carcinoma after liver transplantation from univariate Cox regression analyses (*P* < 0.1)

Variables	Associated with OS		Associated with RFS	
	HR (95% CI)	*P*	HR (95% CI)	*P*
Hypothyroidism	1.95 (1.15, 3.31)	0.014	2.70 (1.27, 5.73)	0.0096
AILD	‐	‐	2.28 (0.95, 5.47)	0.065
Cirrhosis	0.3 (0.06, 1.05)	0.058	‐	‐
TSH	1.05 (1.02, 1.08)	0.0003	1.09 (1.04, 1.13)	<0.0001
NLR	1.07 (1.02, 1.12)	0.005	‐	‐
AFP (per 100 ng/mL)	1.18 (1.03, 1.36)	0.018	‐	‐
CTP score	‐	‐	0.85 (0.71, 1.01)	0.060
MELD score	1.02 (1.00, 1.15)	0.099	‐	‐
Vascular invasion	3.12 (1.83, 5.33)	<0.001	7.30 (3.68, 14.50)	<0.0001
Tumor maximum diameter				
<3 cm	1		1	
≥3 cm	1.77 (1.14, 2.77)	0.012	3.15 (1.62, 6.13)	0.0007
Milan criteria				
Within	1		1	
Beyond	1.76 (1.16, 2.69)	0.0084	3.64 (1.85, 7.16)	0.0002

AFP, alpha‐fetoprotein; AILD, autoimmune liver disease; CTP, Child‐Turcotte‐Pugh; HR, hazard ratio; MELD, model for end‐stage liver disease; NLR, neutrophil to lymphocyte ratio; OS, overall survival; RFS, recurrence‐free survival; TSH, thyroid‐stimulating hormone.

### 3.4 The impact of hypothyroidism on overall survival and recurrence‐free survival

**Figure 1:**
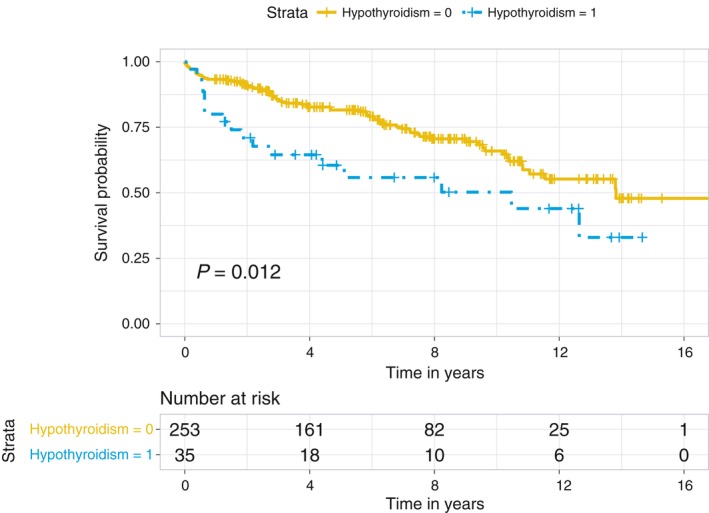
Kaplan‐Meier overall survival curves for hepatocellular carcinoma patients stratified by hypothyroidism

**Figure 2:**
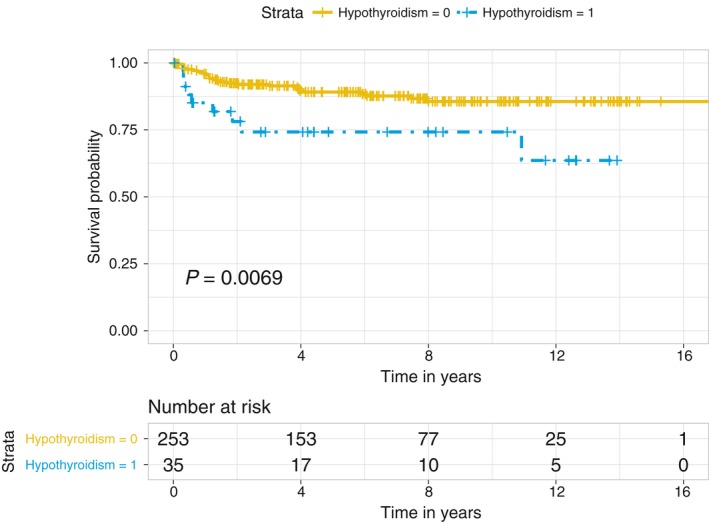
The Kaplan‐Meier recurrence‐free survival curves for hepatocellular carcinoma (HCC) patients stratified by hypothyroidism diagnosed prior to HCC

Figures 1 and 2 show the remarkably lower OS and RFS of patients with hypothyroidism compared to patients without hypothyroidism, respectively. The five‐year OS and RFS rates for HCC patients with hypothyroidism were 60.5% and 74.2%, respectively, compared to 81.6% and 89.1% for those without hypothyroidism; similarly, the ten‐year OS and RFS rates were 50.3% and 74.2% in those with hypothyroidism compared to 66.1% and 85.6% in those without hypothyroidism.

To evaluate the independent impact of hypothyroidism on OS and RFS, we further conducted a multivariate analysis by adjusting for different covariates Table 3. Because TSH level before liver transplantation may reflect the patient's degree of hypothyroidism, we also performed an analysis to evaluate the impact of TSH level on OS and RFS for the entire cohort receiving liver transplantation. Demographic variables adjusted for in model 1 included age, gender, and race/ethnicity. For model 2, in addition to the model 1 variables, we adjusted for variables previously shown to be associated with hypothyroidism, including smoking, obesity, diabetes, hypertension. For model 3, in addition to the model 2 variables, we adjusted for all additional variables with *P* < 0.10 by univariate analysis (AILD, CTP, vascular invasion, tumor diameter, and Milan criteria). The AHRs and corresponding 95% CI for different multivariate models are shown in Table 3. The AHRs of hypothyroidism and TSH in model 3 were 2.45 (1.44, 4.18) and 1.05 (1.02, 1.09), respectively, for OS and 5.54 (2.36, 13.01) and 1.08 (1.03, 1.13) for RFS.

**Table 3:** The impact of hypothyroidism and the TSH level on overall survival and recurrence‐free survival adjusted for different covariates

	Hypothyroidism		Hypothyroidism‐2		TSH	
	AHR	*P*	AHR	*P*	AHR	*P*
OS‐model1	1.78 (1.01, 3.13)	0.047	1.86 (1.15, 3.02)	0.012	1.05 (1.02, 1.08)	0.0006
OS‐model2	1.96 (1.10, 3.49)	0.022	2.05 (1.25, 3.35)	0.004	1.06 (1.03, 1.09)	0.0003
OS‐model3	2.25 (1.18, 4.28)	0.014	2.45 (1.44, 4.18)	0.0009	1.05 (1.02, 1.09)	0.0026
RFS‐model1	2.43 (1.10, 5.35)	0.027	1.93 (0.94, 3.96)	0.073	1.09 (1.04, 1.13)	0.0001
RFS‐model2	3.03 (1.35, 6.77)	0.007	2.23 (1.07, 4.63)	0.033	1.09 (1.05, 1.14)	<0.0001
RFS‐model3	5.54 (2.36, 13.01)	0.0001	3.01 (1.36, 6.64)	0.006	1.08 (1.03, 1.13)	0.0030

OS‐model1 adjusted for age, gender, race/ethnicity; OS‐model2 adjusted for smoking, obesity, diabetes, and hypertension in addition to OS‐model1; and OS‐model3 adjusted for cirrhosis, neutrophil to lymphocyte ratio (NLR), alpha‐fetoprotein (AFP), model for end‐stage liver disease (MELD) score, tumor diameter, vascular invasion, and Milan criteria in addition to OS‐model2. RFS‐model1 adjusted for age, gender, race/ethnicity; RFS‐model2 adjusted for smoking, obesity, diabetes, and hypertension in addition to RFS‐model1; and RFS‐model3 adjusted for autoimmune liver disease (AILD), Child‐Turcotte‐Pugh (CTP) score, tumor diameter, vascular invasion, and Milan criteria in addition to RFS‐model2.

AHR, adjusted hazard ratio; OS, overall survival; RFS, recurrence‐free survival; TSH, thyroid‐stimulating hormone.

**Figure 3:**
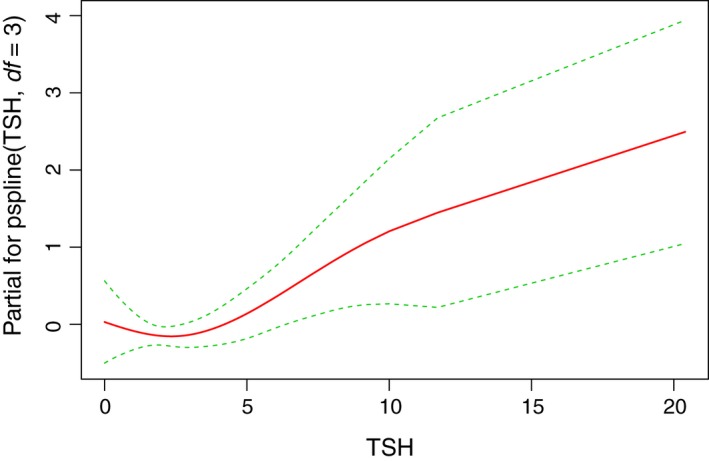
Impact of thyroid‐stimulating hormone (TSH) before liver transplantation on risk of death. The trend didn't change after adjusting for other risk factors, including age, gender, race/ethnicity, smoking, obesity, diabetes, hypertension, cirrhosis, neutrophil to lymphocyte ratio (NLR), alpha‐fetoprotein (AFP), model for end‐stage liver disease (MELD) score, tumor diameter, vascular invasion, Milan criteria. The adjusted hazard ratio for TSH >2.5 mIU/L is 1.05 (1.02, 1.09; *P* = 0.0012)

Figure 3 shows the spline curve of the probability of death as the TSH increases. The result shows the same trend after adjusting for other factors included in model 3 of Table 3 (n = 273). The adjusted spline curves suggested a cutoff value separating low‐ and high‐risk groups at 2.5 mIU/L. The AHR for TSH lower vs higher than 2.5 mIU/L was 0.89 (0.63, 1.25) and 1.05 (1.02, 1.09), respectively. Due to the limited number of recurrence events (n = 37), we were unable to perform the spline curve analysis to evaluate the relationship of TSH with recurrence.

**Figure 4:**
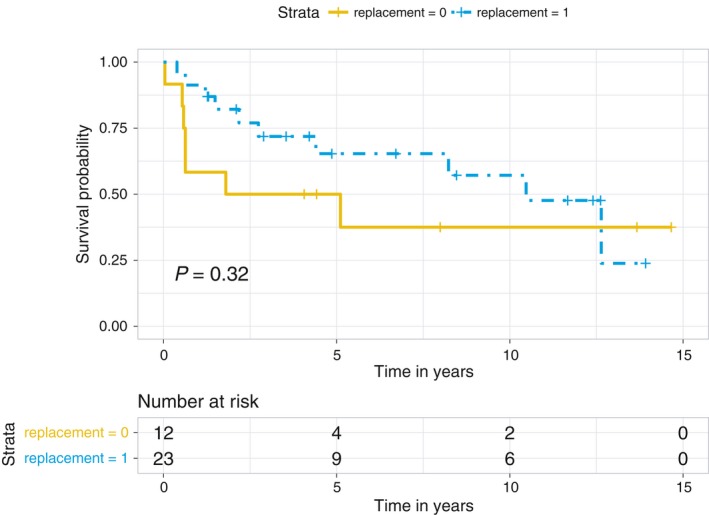
Kaplan‐Meier overall survival curves stratified by use of replacement therapy for hypothyroidism (*P* = 0.32). The hazard ratio (HR) of replacement therapy from univariate analysis is 0.61 (0.23, 1.70)

From the overall survival curve Figure 4, it appeared that the patients with hypothyroidism who received thyroid hormone replacement had a better overall outcome, and the HR from the univariate analysis was 0.61 (0.23, 1.70). The large range for the 95% confidence interval is likely due to the small size of the group (n = 35).

### 3.5 Stratified analysis of the hazard ratios of hypothyroidism in different subgroups

**Figure 5:**
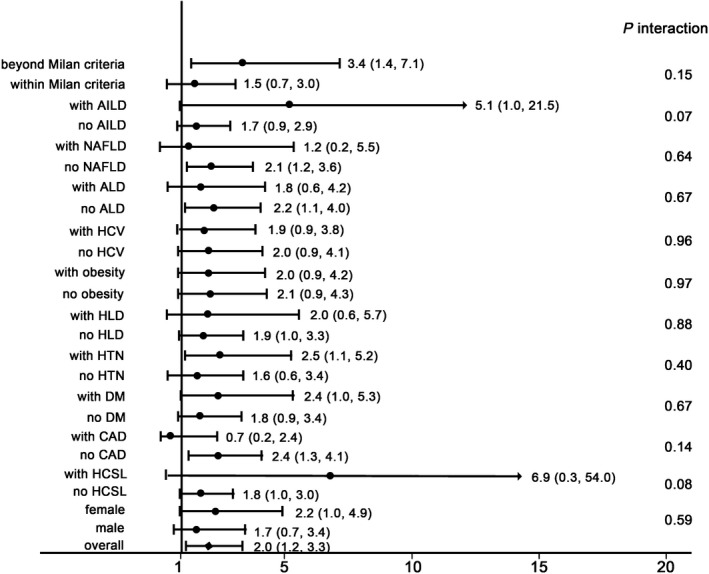
Subgroup analysis and interaction test of the effects of hypothyroidism on overall survival of hepatocellular carcinoma patients. AILD, autoimmune liver disease; NAFLD, nonalcoholic fatty liver disease; ALD, alcoholic liver disease; HCV, hepatitis C virus; HLD, hyperlipidemia; HTN, hypertension; DM, diabetes mellitus; CAD, coronary artery disease; HCSL, hypercholesterolemia

We further analyzed the impact of hypothyroidism in different subgroups across gender, underlying liver diseases, comorbidities, and pathological Milan criteria. The HRs and *P* value for interaction test were shown in Figure 5.

Comparing the different subgroups, hypothyroidism was associated with a higher risk of death than the overall HR in the following subgroups: (a) patients beyond Milan criteria based on the pathological explant report (HR = 3.36, 95% CI = 1.41, 7.14); (b) patients with autoimmune liver disease (AILD; HR = 5.12, 95% CI = 1.03, 21.45); (c) patients without NAFLD (HR = 2.10, 95% CI = 1.15, 3.59); (d) patients without ALD (HR = 2.16, 95% CI = 1.08, 4); (e) patients with hypertension (HTN; HR = 2.48, 95% CI = 1.13, 5.15); and (f) patients without coronary artery disease (CAD; HR = 2.36, 95% CI = 1.26, 4.12). However, the *P* values for the interaction tests among the stratified groups were not significant.

### 3.6 Sensitivity analyses for subgroups

Sensitivity analyses were conducted to clarify the impact of hypothyroidism on different cohorts, and multivariate Cox analyses were conducted by adjusting for the potential confounders identified in model 3 Table 2.

After excluding patients who died within 1 month after transplantation (n = 5), the AHR of hypothyroidism was 2.31 (1.23, 4.32) for OS and 2.02 (1.03, 3.98) for RFS. When the analysis was conducted after including the additional 30 pre‐diagnosed HCC patients with TSH >5 mIU/L prior to liver transplantation, the AHR was 2.45 (1.44, 4.18) for OS and 3.01 (1.36, 6.64) for RFS Hypothyroidism‐2 in Table 2. Finally, we performed the analysis on the overall cohort (n = 343), and the results remained consistently significant with an AHR of 2.48 (1.39, 4.43) for OS and 2.27 (1.28, 4.03) for RFS.

## 4 DISCUSSION

This is the first study focusing on the relationship between hypothyroidism and outcomes of HCC patients after liver transplantation. Our study revealed that hypothyroidism was an independent risk factor associated with poor overall survival and recurrence‐free survival after liver transplantation. Similar findings were found for patients with high TSH. Plotting a spline curve of TSH value with the adjusted hazard ratio, the risk of death increases with TSH values exceeding 2.5 mIU/L. It is, however, not clear whether managing hypothyroidism to a TSH value than 2.5 mIU/L prior to liver transplantation would improve outcomes.

The impact of hypothyroidism on overall survival does not change with gender, obesity, HCV infection, or DM. Stratified analyses show that hypothyroidism predicts a nonsignificant trend toward poor survival in HCC patients with hypertension and underlying AILD and in patients without CAD, NAFLD, and ALD.

The basic results persisted after excluding patients incidentally diagnosed with HCC at the time of transplantation and also after excluding patients who died within 1‐month post‐liver transplantation. When patients with a single abnormal high TSH measurement and consequent uncertain diagnosis of hypothyroidism were assumed to have hypothyroidism, the observed association persisted.

Why hypothyroidism causes poor survival and higher recurrence is not clear. The survival curve shown in Figure 1 shows that the largest difference between the hypothyroid and non‐hypothyroid groups occurred in the early years after liver transplantation. Based on previous publications 14, 15, 17, we can speculate that hypothyroidism may be a factor that accelerates the malignant process of HCC. Even though its role in other cancers is controversial, the effects of hypothyroidism on the incidence and outcomes of HCC are consistent. Downregulation of thyroid hormone receptor (TR) β1 and, to a lesser extent, TRα1 was observed only in the preneoplastic lesions positive for the progenitor cell marker, cytokeratin‐19 (Krt‐19), and HCC of the resistant‐hepatocyte rat model, suggesting that a hypothyroid status favors the onset and progression of preneoplastic lesions to HCC. TRβ1 downregulation was also identified in the vast majority of analyzed human HCCs, compared to the matched peri‐tumorous liver or to normal liver. The possible mechanism may be associated with an increased expression of TRβ1‐targeting miR‐181a, which was found in human cirrhotic peritumoral tissue, compared to normal liver. T3 treatment can induce upregulation of TRβ1 and was associated with nodule regression.^15^

While the frequencies of hypertension and obesity were higher among HCC patients with hypothyroidism, they were not independently predictive of worse outcome. The consistent observation of an association of hypothyroidism with risk and outcomes of HCC calls for additional investigation of the biologic mechanisms underlying these observations.

Overall, our study identifies hypothyroidism as a potentially important factor in assessing risk and outcomes of HCC patients within the transplantation setting. Limitations should be considered for this study. The retrospective and single‐center cohort limited the sample size and enough useful information, selection, and measurement bias may exist. Another limitation is that from the current data, it's hard to speculate the direct mechanism between hypothyroidism and the worse outcome. From the list of death reason Table S2, more HCC patients with hypothyroidism die of renal failure and respiratory failure besides HCC recurrence. Therefore, additional cohort and multicenter studies are warranted to validate these observations and reveal the possible mechanism of this novel finding.

## CONFLICT OF INTEREST

Nothing to disclose.
