# Supplementary material for: Hypothyroidism is associated with worse outcomes of hepatocellular carcinoma patients after liver transplantation
Source: Cancer Med. 2018 Nov 19;7(12):5870–8. doi: 10.1002/cam4.1797 (PMC6308061; doi:10.1002/cam4.1797)
Supplement: Supplementary file 2 [file CAM4-7-5870-s002.docx]

Supplemental table 1. Baseline characteristics of study participants before liver transplantation

| **Variables** | **Incidental HCC**  **n=55** | **Pre-diagnosed HCC**  **n=288** | **p** |
| --- | --- | --- | --- |
| Gender = Male (%) | 38 (69.1) | 210 (72.9) | 0.677 |
| Age (median [IQR]) | 57.36 [52.84, 61.53] | 58.43 [54.74, 63.12] | 0.317 |
| CAD = yes (%) | 11 (20.0) | 49 (17.0) | 0.733 |
| HCSL = yes (%) | 7 (12.7) | 23 (8.0) | 0.379 |
| DM = yes (%) | 29 (52.7) | 114 (39.6) | 0.096 |
| HTN = yes (%) | 21 (38.2) | 103 (35.8) | 0.850 |
| HLD = yes (%) | 12 (21.8) | 55 (19.1) | 0.779 |
| Hypothyroidism = yes (%) | 5 (9.1) | 35 (12.2) | 0.675 |
| Obesity = yes (%) | 26 (47.3) | 113 (39.2) | 0.336 |
| Race = White (%) | 48 (94.1) | 234 (88.0) | 0.299 |
| Smoking (%) |  |  | 0.014 |
| current | 4 (7.3) | 71 (24.7) |  |
| former | 19 (34.5) | 71 (24.7) |  |
| never | 32 (58.2) | 146 (50.7) |  |
| HCV = yes (%) | 18 (32.7) | 150 (52.1) | 0.013 |
| ALD = yes (%) | 15 (27.3) | 92 (31.9) | 0.599 |
| NAFLD = yes (%) | 14 (25.5) | 53 (18.4) | 0.306 |
| HBV = yes (%) | 1 (1.8) | 20 (6.9) | 0.252 |
| AILD = yes (%) | 8 (14.5) | 26 (9.0) | 0.313 |
| Other etiology = yes (%) | 7 (12.7) | 20 (6.9) | 0.236 |
| AFP (median [IQR]) | 3.70 [2.50, 8.65] | 8.00 [4.10, 24.25] | <0.001 |
| CTP score (mean (sd)) | 10.05 (1.66) | 7.60 (2.18) | <0.001 |
| MELD sore (median [IQR]) | 23.00 [19.00, 29.50] | 12.00 [9.00, 18.00] | <0.001 |
| Final status = deceased (%) | 13 (23.6) | 88 (30.6) | 0.384 |
| Recurrence = yes (%) | 0 (0.0) | 37 (12.8) | 0.010 |

BMI, body mass index; CAD, coronary artery disease; HCSL, hypercholesterolemia; DM, diabetes mellitus; HTN, hypertension; HLD, hyperlipidemia; HCV hepatitis C virus; ALD, alcoholic liver disease; NAFLD, nonalcoholic fatty liver disease; HBV, hepatitis B virus; AILD, autoimmune liver disease; TSH, thyroid-stimulating hormone; MELD, model for end-stage liver disease.

Supplemental table 2. Death reason of patients grouped by hypothyroidism

| **Death reason N(%)** | **No hypothyroidism**  **N = 253** | **Hypothyroidism**  **N = 35** |
| --- | --- | --- |
| Final status = deceased (%) | 71 (28.1) | 17 (48.6) |
| Recurrence | 24 (33.8) | 7 (41.2) |
| Infection | 7 (9.9) | - |
| Graft dysfunction | 5* (7) | 1 (5.9) |
| Operation related | 6 (8.5) | 1 (5.9) |
| Other cancer | 6 (8.5) | 1 (5.9) |
| Respiratory failure | 2 (2.8) | 1 (5.9) |
| Renal failure | - | 3 (17.6) |
| Emergent asystole | 1 (1.4) | - |
| PTLD | 1 (1.4) | - |
| Recurrent HCV | 1 (1.4) | - |
| Unknown | 17 (23.9) | 3 (17.6) |
| Defib disorder | 1 (1.4) | - |

PTLD, Posttransplant lymphoproliferative disease. *One patient in this group also had PTLD.
